# Supplementary material for: Porphyromonas gingivalis aggravates colitis via a gut microbiota-linoleic acid metabolism-Th17/Treg cell balance axis
Source: Nat Commun. 2024 Feb 22;15:1617. doi: 10.1038/s41467-024-45473-y (PMC10883948; doi:10.1038/s41467-024-45473-y)
Supplement: Supplementary file 1 — Supplementary Information [file 41467_2024_45473_MOESM1_ESM.pdf]

Supplementary Information for

Porphyromonas gingivalis aggravates colitis via a gut microbiota-linoleic acid metabolism-Th17/Treg balance axis

Lu Jia[1], Yiyang Jiang[1], Lili Wu[1], Jingfei Fu[1], Juan Du[1], Zhenhua Luo[1], Lijia Guo[2], Junji Xu[1#], Yi Liu[1#]

1 Laboratory of Tissue Regeneration and Immunology and Department of Periodontics, Beijing Key Laboratory of Tooth Regeneration and Function Reconstruction, School of Stomatology, Capital Medical University, Beijing, P. R. China
2 Department of Orthodontics School of Stomatology, Capital Medical University, Beijing, P. R. China.

# Co-corresponding author. Tel.: +86 10 57099450; Fax: +86 10 57099450.
E-mail addresses: uujkl@163.com (J Xu) & lililiuyi@163.com (Y Liu)

SUPPLEMENTARY FIGURES

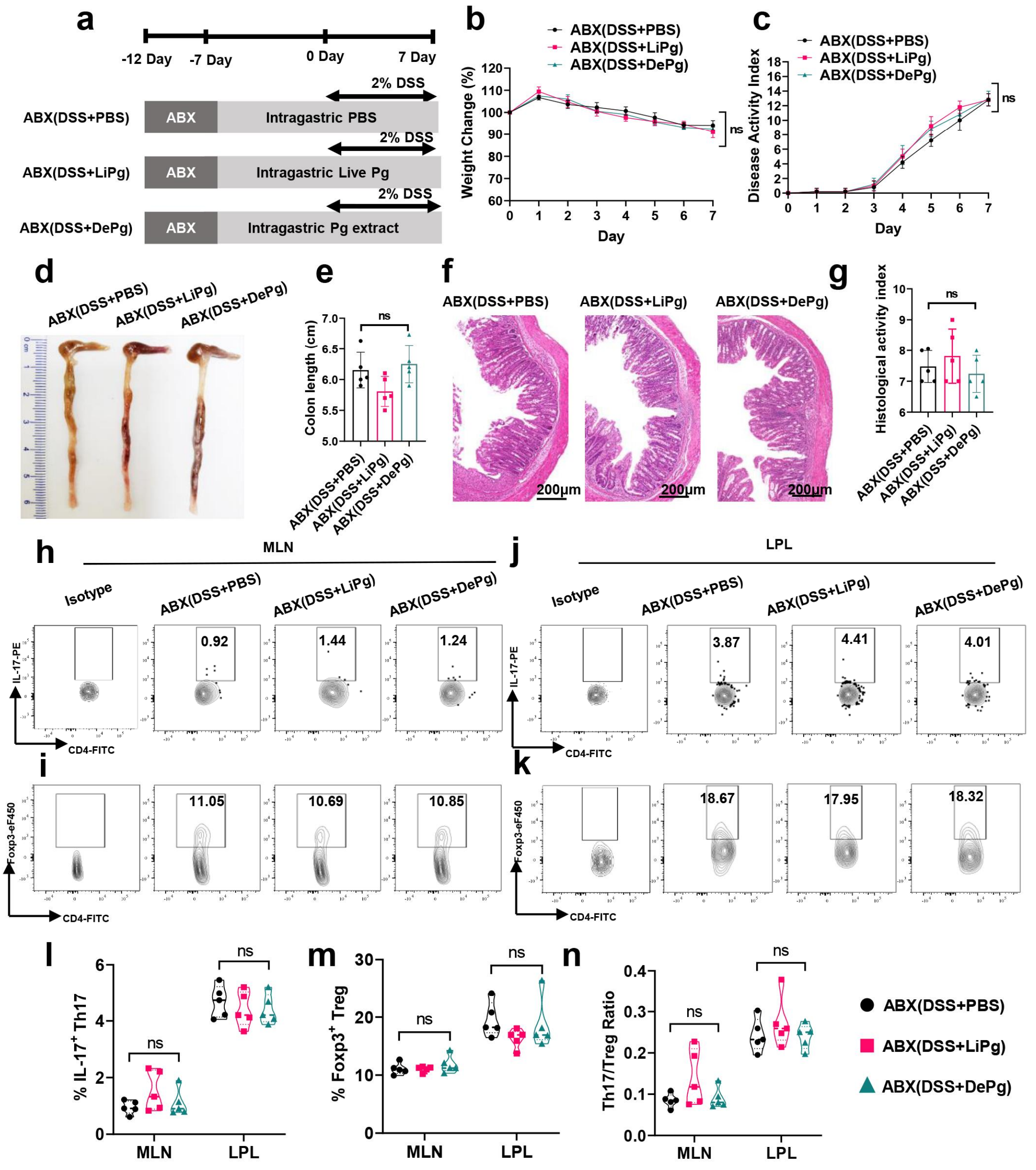

Supplementary Fig. 1. The aggravation of dietary Pg in colitis mice and the Th17/Treg imbalance disappeared after depletion of the gut microbiota. (**a**) Diagram of the gut microbiota depletion experiment. WT mice were administered a course of intragastric antibiotics for five days for gut microbiota depletion before DSS treatment. The ABX(DSS+PBS), ABX(DSS+LiPg), and ABX(DSS+DePg) mice were gavaged with PBS, live Pg, or ultrasonic extract of Pg, respectively. (**b**) Body weight of ABX(DSS+PBS), ABX(DSS+LiPg) and ABX(DSS+DePg) mice during DSS-induced colitis, n=5 independent samples. (**c-g**) Disease activity index (DAI) (**c**), colon length (**d-e**), and histological score (**f-g**) of ABX(DSS+PBS), ABX(DSS+LiPg), and ABX(DSS+DePg) mice on day 8 after DSS induction, n=5 independent samples. Scale

bar, 200 µm. (**h-k**) The proportions of IL-17^{+} Th17 cells and Foxp3^{+} Treg cells and their ratio among total CD4^{+} T cells within the MLNs and LPLs were detected by flow cytometry and statistically analysed (**l-n**), n=5 independent samples. Data are presented as the mean ± SEM; ns: no significant difference by two-tailed one-way ANOVA. Pg, *Porphyromonas gingivalis*; MLN, mesenteric lymph node cells; LPL, colon lamina propria lymphocytes; ANOVA, analysis of variance; DSS, dextran sodium sulfate. Source data are provided as a Source Data file.

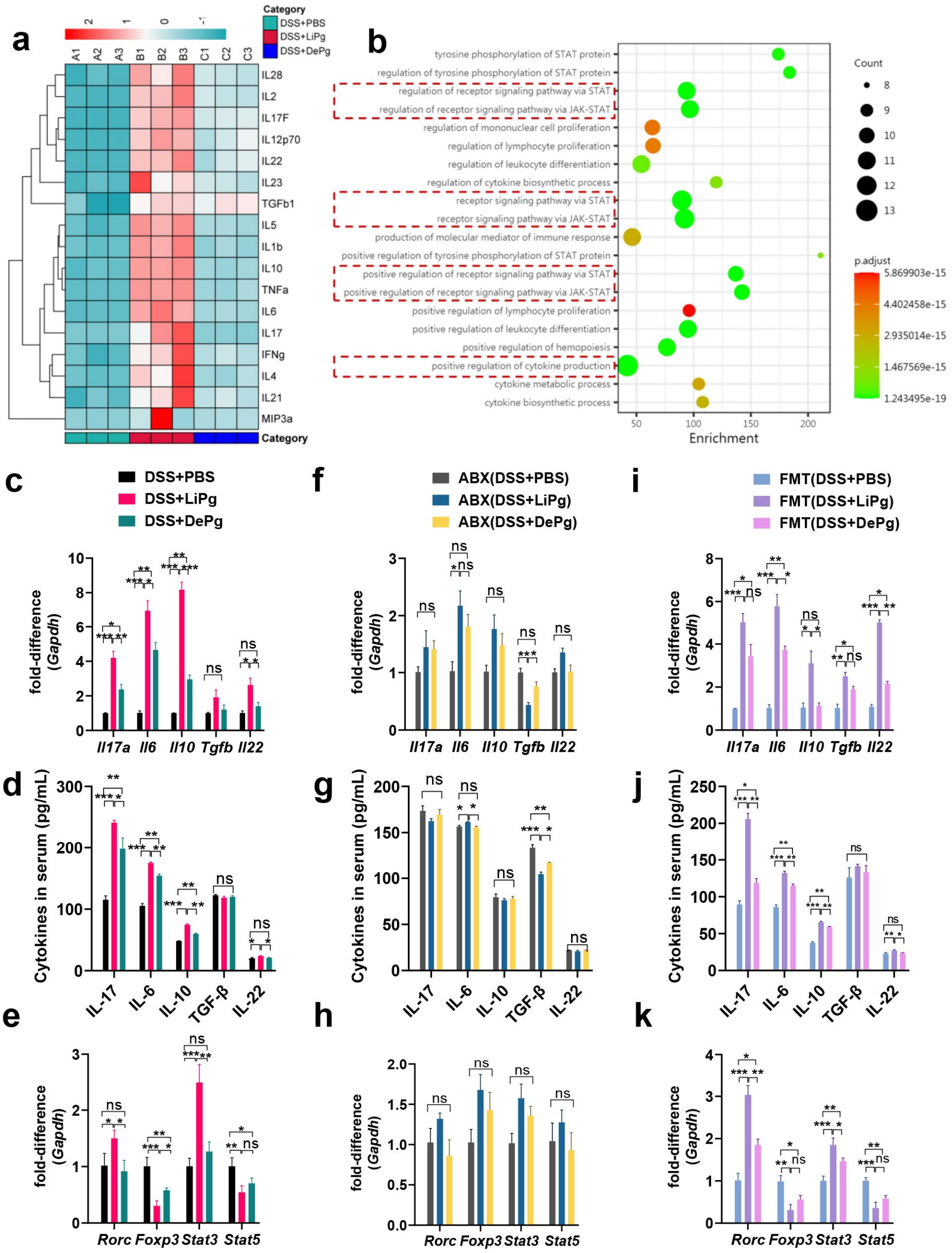

Supplementary Fig. 2. Oral administration of Pg altered the cytokine profile in colitis mice in a gut microbiota-dependent manner. (**a**) Heatmap representation of cytokine levels from DSS+PBS, DSS+LiPg, and DSS+DePg colon tissue supernatants as measured by Quantibody cytokine array. n=3 independent samples. The raw mean fluorescence intensity of all proteins was log2 transformed before clustering. (**b**) KEGG enrichment pathway analysis was performed to identify pathways that play a key role in regulating Th17 differentiation. (**c** and **e**) *IL-17A*, *IL-6*, *IL-10*, *TGF-β*, and *IL-22* gene expression and cytokine levels in colon tissue homogenate were measured by RT–qPCR and ELISA in the DSS+PBS, DSS+LiPg, and DSS+DePg groups. n=3 independent samples. (**f** and **h**) *IL-17A*, *IL-6*, *IL-10*, *TGF-β*, and *IL-22* gene expression and cytokine

levels in colon tissue homogenate were measured by RT–qPCR and ELISA in the ABX(DSS+PBS), ABX(DSS+LiPg), and ABX(DSS+DePg) groups. n=3 independent samples. (**i** and **k**) *IL-17A*, *IL-6*, *IL-10*, *TGF-β*, and *IL-22* gene expression and cytokine levels in colon tissue homogenate were measured by RT–qPCR and ELISA among the FMT (DSS+PBS), FMT (DSS+LiPg), and FMT (DSS+DePg) groups. n=3 independent samples. (**d, g, j**) *Rorc*, *Foxp3*, *Stat3*, and *Stat5* gene expression in colon tissue homogenate among DSS+PBS, DSS+LiPg, and DSS+DePg groups and ABX(DSS+PBS), ABX(DSS+LiPg), and ABX(DSS+DePg) groups as well as FMT(DSS+PBS), FMT(DSS+LiPg), and FMT(DSS+DePg) groups were measured by RT–qPCR. n=3 independent samples. Data are presented as the mean ± SEM; *p<0.05; **p<0.01; ***p<0.001; ns: no significant difference by two-tailed one-way ANOVA. Source data are provided as a Source Data file.

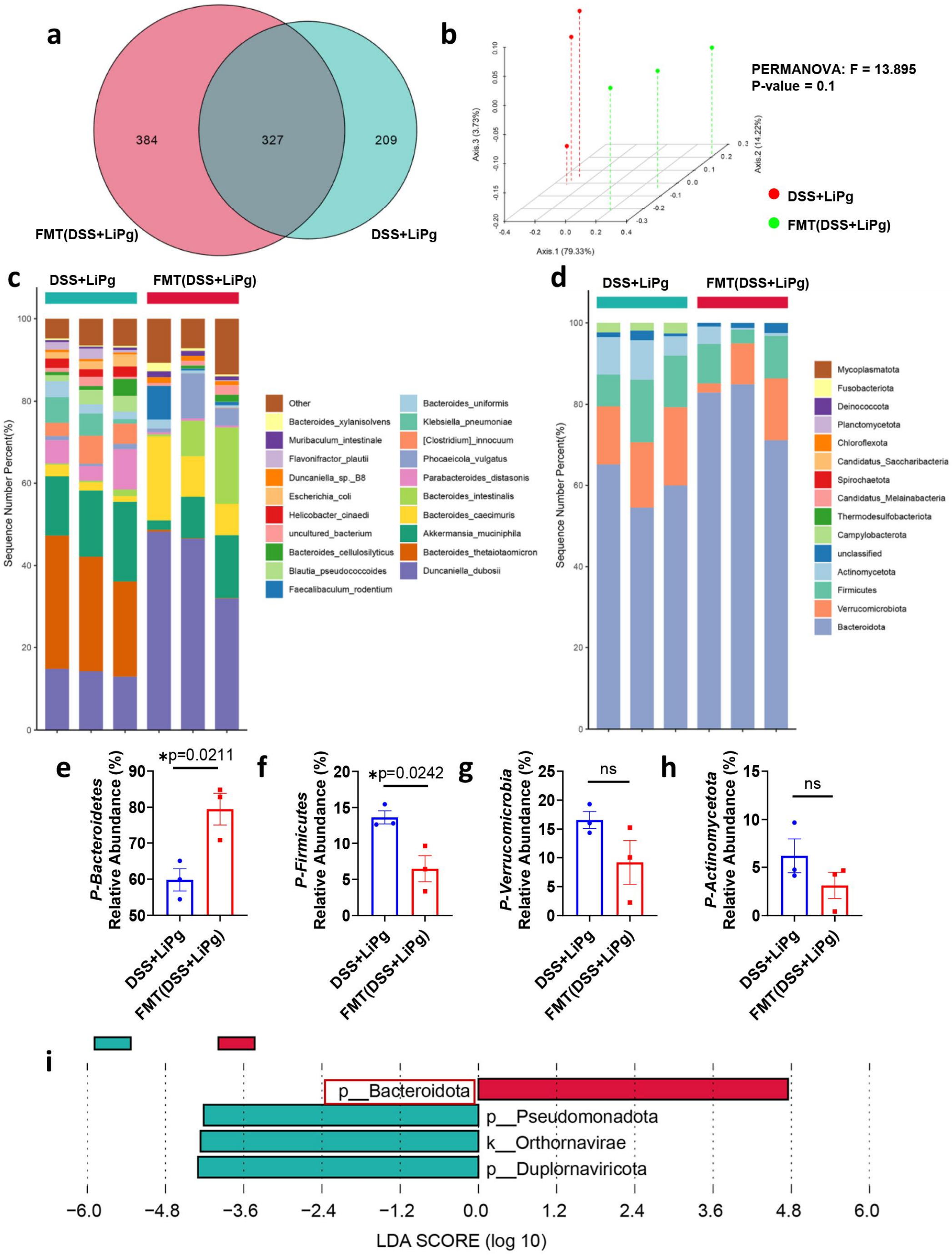

Supplementary Fig. 3. The reconstruction of donor microbiota after FMT. (**a**) High-throughput metagenomic sequencing of the faecal bacterial genomes from DSS+LiPg and FMT (DSS+LiPg) mice. n=3 independent samples. The number of common or unique species between the two groups in the Venn diagram. (**b**) 3D PCoA graph based on the Bray–Curtis's distance matrix. PERMANOVA: F=13.895, P value=0.1. PERMANOVA: permutational multivariate analysis of variance. (**c**) Column diagram of the relative distribution of each sample at the species level (top 20). (**d**) Column diagram of the relative distribution of each sample at the phylum level (top 10). The Y-axis is the sequence number percent, indicating the ratio of this phylum level to

the total annotation data. (**e-h**) The box plots indicate the top 4 relative abundances of each bacterial group at the phylum level (*Bacteroidetes*, *Firmicutes*, *Verrucomicrobia*, and *Actinobacteria*). n=3 independent samples. *$p<0.05$ by two-tailed unpaired t-test. (**i**) The most differentially abundant taxa of characteristic microorganisms at the phylum level between the DSS+LiPg and FMT (DSS+LiPg) group mice based on ANCOM (LDA Sore=4 by LEfSe). LDA Score: linear discriminant analysis score. ANCOM: analysis of composition of microbiomes. Source data are provided as a Source Data file.

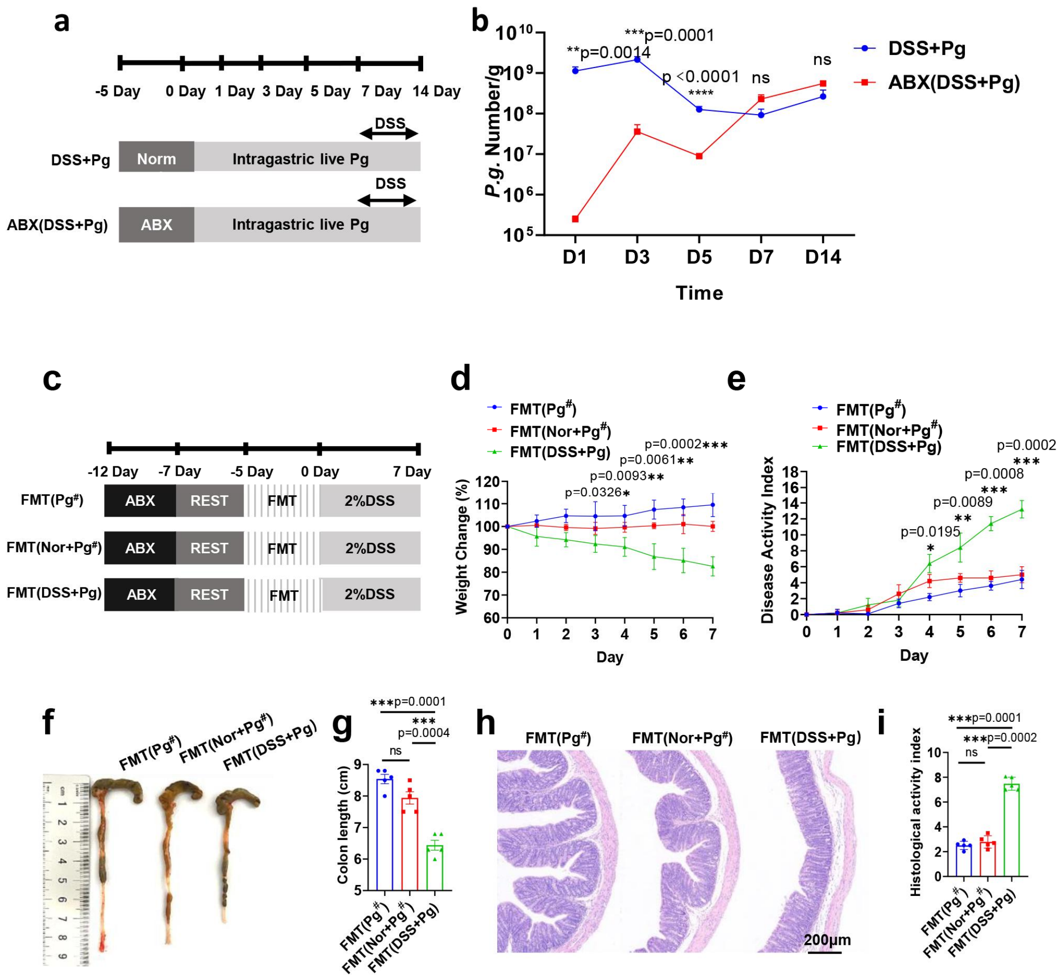

Supplementary Fig. 4. The aggravation of colitis after FMT was a consequence of the whole gut microbiota, not the transfer of Pg only. (**a**) WT mice were given by gavage of the quadruple antibiotic cocktails for gut microbiota depletion (ABX(DSS+Pg) group). A total of 1×10^9 CFUs of live Pg suspended in 100 μL PBS was given to each mouse by gavage through a feeding needle every other day for 14 days. DSS was administered from the 7th day. (**b**) Line chart of Pg absolute copies per gram of mice feces in both groups by qPCR. n=5 independent samples. Data are presented as the mean ± SEM; **p<0.01; ***p<0.001 by two-tailed t-test. (**c**) Diagram of microbiota transplantation experiment. ABX: antibiotic cocktail. FMT: fecal microorganism transplantation. Pg$^{\#}$, the actual content of Pg in the feces of DSS+LiPg group mice. Transplantation of the Pg$^{\#}$/fecal suspension of Nor+Pg$^{\#}$/feces of DSS+Pg group into recipient mice→the FMT(Pg$^{\#}$/Nor+Pg$^{\#}$/DSS+Pg) group. (**d**) Body weight of FMT(Pg$^{\#}$), FMT(Nor+Pg$^{\#}$), and FMT(DSS+Pg) mice during DSS-induced colitis, n=5 independent samples. (**e-g**) Disease Activity Index (DAI) (**e**), colon length (**f-g**), and histological score (**h-i**) of three group mice on day 8 after DSS induction, n=5 independent samples. Scale bar, 200 μm. Data are presented as the mean ± SEM; *p<0.05; **p<0.01; ***p<0.001 by two-tailed one-way ANOVA. Pg, *Porphyromonas gingivalis*. Source data are provided as a Source Data file.

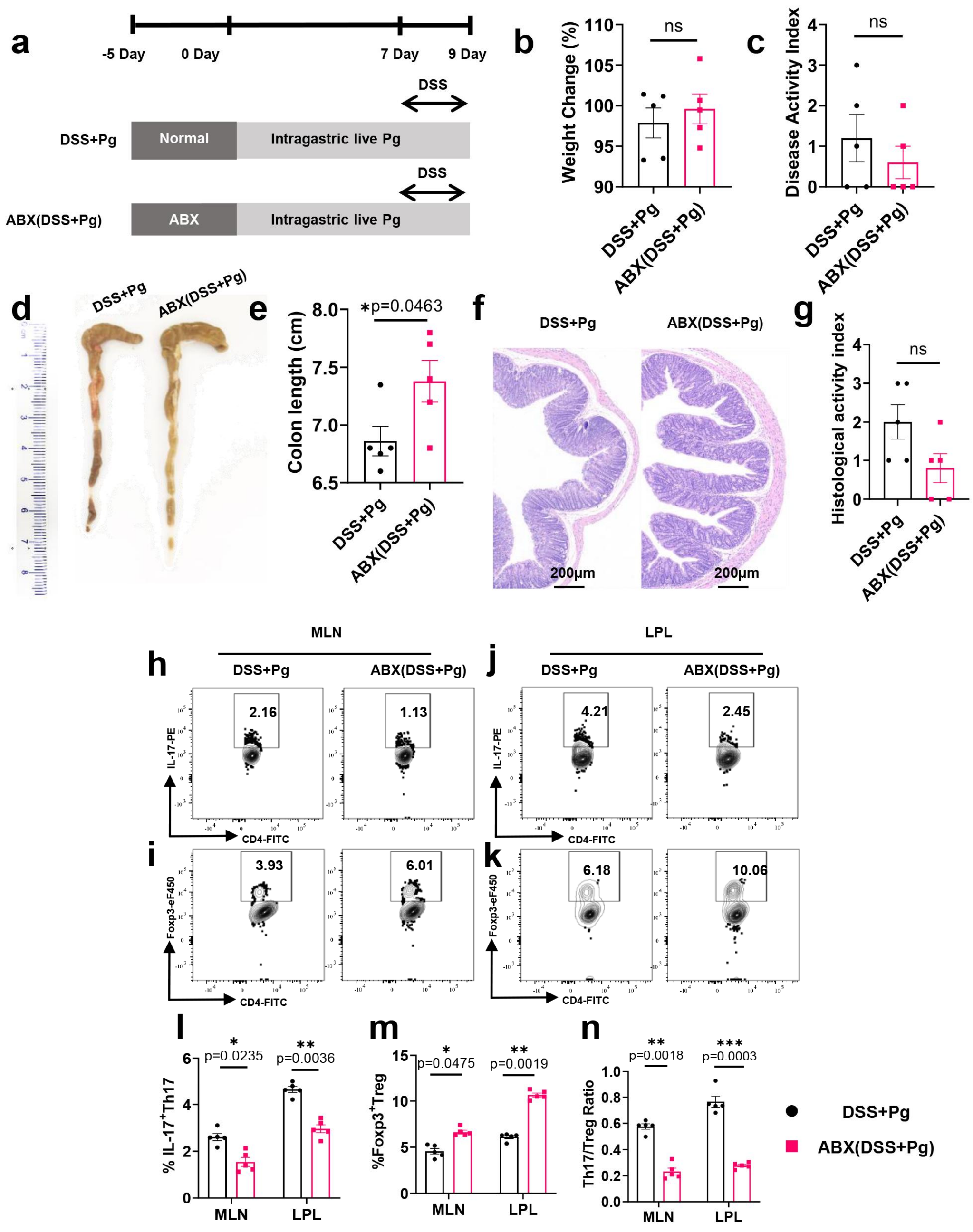

Supplementary Fig. 5. Increased Th17/Treg ratio happened at the early stage during the exacerbation of colitis by gavage of Pg. (**a**) Diagram of this experiment. WT mice were administrated with antibiotics cocktail for five days to gut microbiota depletion before Pg and DSS treatment named the DSS+Pg group and the ABX(DSS+Pg) group. Mice were gavaged with Pg for nine days, with the last two days of treatment with 3.0% DSS. (**b**) Body weight of the DSS+Pg group and ABX(DSS+Pg) group mice during DSS-induced colitis, n=5 independent samples. (**c-g**) DAI, colon length, and HAI of the DSS+Pg group and ABX(DSS+Pg) group mice on day 2 after DSS induction, n=5 independent samples. Scale bar, 200 μm. (**h-k**) The proportions of IL-17^{+}Th17 cells, Foxp3^{+}Treg cells, and their ratio among total CD4^{+}T cells within

MLN and LPL were detected by flow cytometry and statistically analyzed (**l-n**), n=5 independent samples. Data are presented as the mean ± SEM; *p<0.05; **p<0.01; ***p<0.001 by two-tailed unpaired t-test. Pg, *Porphyromonas gingivalis*; MLN, mesenteric lymph nodes; LPL, lamina propria lymphocytes. Source data are provided as a Source Data file.

a

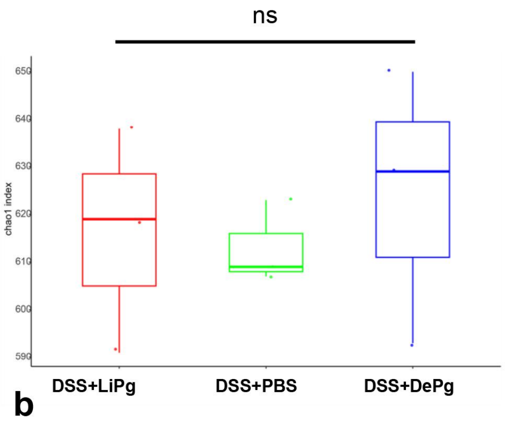

d

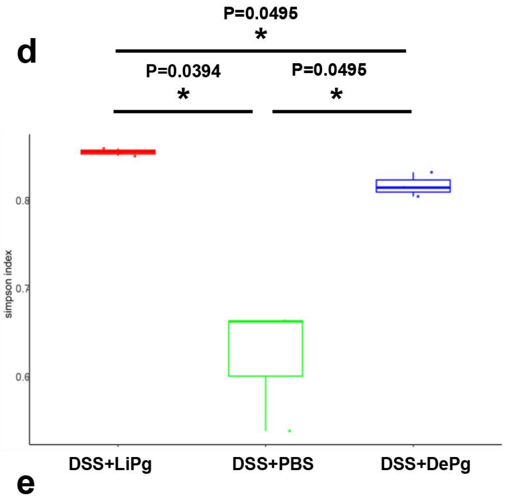

b

Kruskal-Wallis (all groups)	
H	p-value
0.62	**0.73263246**

e

Kruskal-Wallis (all groups)	
H	p-value
7.2	**0.027323722**

c

Kruskal-Wallis (pairwise)			
Group 1	Group 2	H	p-value
DSS+DePg (n=3)	DSS+LiPg (n=3)	0.428571	**0.512691**
DSS+DePg (n=3)	DSS+PBS (n=3)	0.428571	**0.512691**
DSS+LiPg (n=3)	DSS+PBS (n=3)	0.047619	**0.827259**

f

Kruskal-Wallis (pairwise)			
Group 1	Group 2	H	p-value
DSS+DePg (n=3)	DSS+LiPg (n=3)	3.857142857	**0.049534613**
DSS+DePg (n=3)	DSS+PBS (n=3)	3.857142857	**0.049534613**
DSS+LiPg (n=3)	DSS+PBS (n=3)	4.929554356	**0.039435622**

g

Bray Curtis Distance	DSS+DePg			DSS+PBS			DSS+LiPg		
DSS+DePg	0	0.090936323	0.27880662	0.330945219	0.420623184	0.335337772	0.46959413	0.472537263	0.4343
	0.090936323	0	0.20552637	0.324882784	0.414938624	0.323210775	0.44009689	0.439524272	0.41211
	0.27880662	0.20552637	0	0.342224994	0.424120943	0.32483115	0.42466455	0.422127915	0.40369
DSS+PBS	0.330945219	0.324882784	0.342224994	0	0.146510605	0.063232918	0.54307821	0.53407748	0.51023
	0.420623184	0.414938624	0.424120943	0.146510605	0	0.154772023	0.57091756	0.503028316	0.49689
	0.335337772	0.323210775	0.32483115	0.063232918	0.154772023	0	0.53486997	0.531369699	0.51037
DSS+LiPg	0.469594133	0.440096886	0.424664549	0.54307821	0.570917565	0.534869973	0	0.189908384	0.13219
	0.472537263	0.439524272	0.422127915	0.53407748	0.503028316	0.531369699	0.18990838	0	0.10601
	0.434304611	0.412112299	0.40368747	0.51022863	0.496890268	0.510374442	0.1321912	0.106008963	0

	Mean	SD
DSS+DePg *vs* DSS+PBS	0. 360123938	0. 042683135
DSS+LiPg *vs* DSS+PBS	0. 526092731	0. 021984591
DSS+DePg *vs* DSS+LiPg	0. 435405489	0. 022181285

Supplementary Fig. 6. The alpha diversity analysis and the Bray-Curtis distance matrix of PCoA analysis of the fecal bacteria. (**a**) The Chao1 boxplot to calculate the community richness based on the number of OTUs from the DSS+PBS, DSS+LiPg, and DSS+DePg group mice feces. (**b-c**) The p-value of all groups or pairwise of the Chao1 index after the Kruskal-Wallis's test. (**d**) The Simpson boxplot to calculate the community diversity from the three group mice feces. (**e-f**) The p-value of all groups or pairwise of the Simpson index after the Kruskal-Wallis's test. p＜0.05, significant difference. (**g**) The Bray-Curtis distance matrix of PCoA analysis of the fecal bacteria from the DSS+PBS, DSS+LiPg, and DSS+DePg group mice. The smaller the sample distance between groups, the greater the similarity between groups.

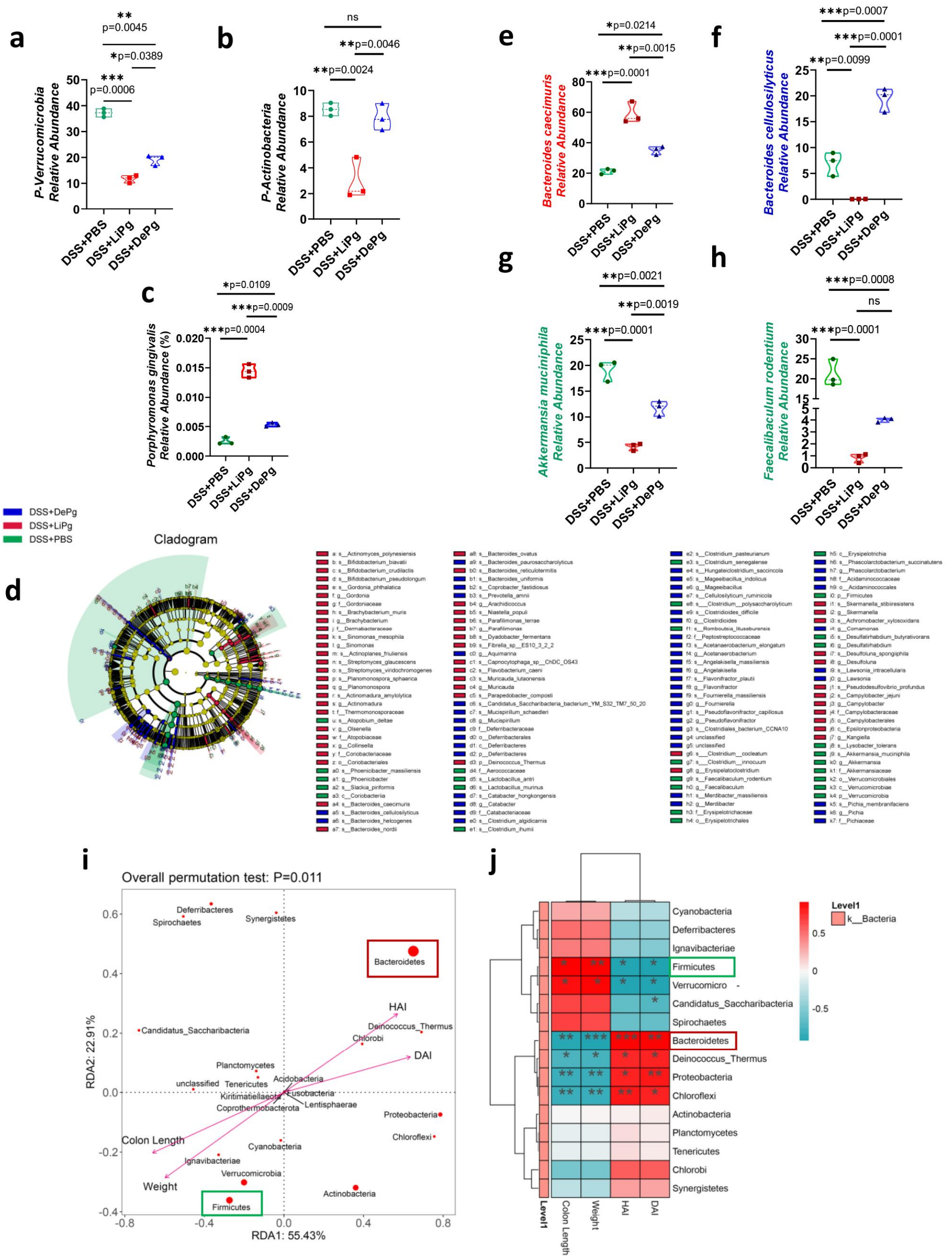

Supplementary Fig. 7. Oral administration of Pg altered the gut microbiota composition. (**a** and **b**) The box plots indicate the relative abundances of each bacterial group at the phylum level (*Verrucomicrobia* and *Actinobacteria*). n=3 independent samples. (**c**) The box plots indicate each bacterial group's relative abundance of *Porphyromonas gingivalis* (Pg). n=3 independent samples. (**d**) Taxonomic cladogram from LEfSe (LDA Sore=2), depicting the taxonomic association between microbiome communities from the DSS+PBS, DSS+LiPg, and DSS+DePg groups. Each node represents a specific taxonomic type. (**e-h**) The most differentially abundant taxa of characteristic microorganisms at the species level from DSS+PBS, DSS+LiPg, and

DSS+DePg mice (LDA Sore=4 by LEfSe) are indicated by box plots. (**i**) RDA ranking diagram at the phylum level. Arrows represent environmental factors, and the length of the arrow line represents the degree of correlation between the environmental factor, community distribution, and species distribution (explaining the magnitude of variance). The longer the arrow, the more significant the correlation. Each point represents a phylum; the more significant the point, the higher the phylum's abundance. The angle between the arrow line and the point represents the correlation between a specific environmental factor and the phylum. The smaller the angle, the higher the correlation. Acute angle: positive correlation; Obtuse angle: negative correlation; Right angle: no correlation. (**j**) The correlation heat maps between the phyla and environmental factors. The X-axis represents environmental factors, while the Y-axis represents phyla. Red represents a positive correlation, while blue represents a negative correlation. *$0.01 \leqslant p < 0.05$; **$0.001 \leqslant p < 0.01$; ***$p < 0.001$. Source data are provided as a Source Data file.

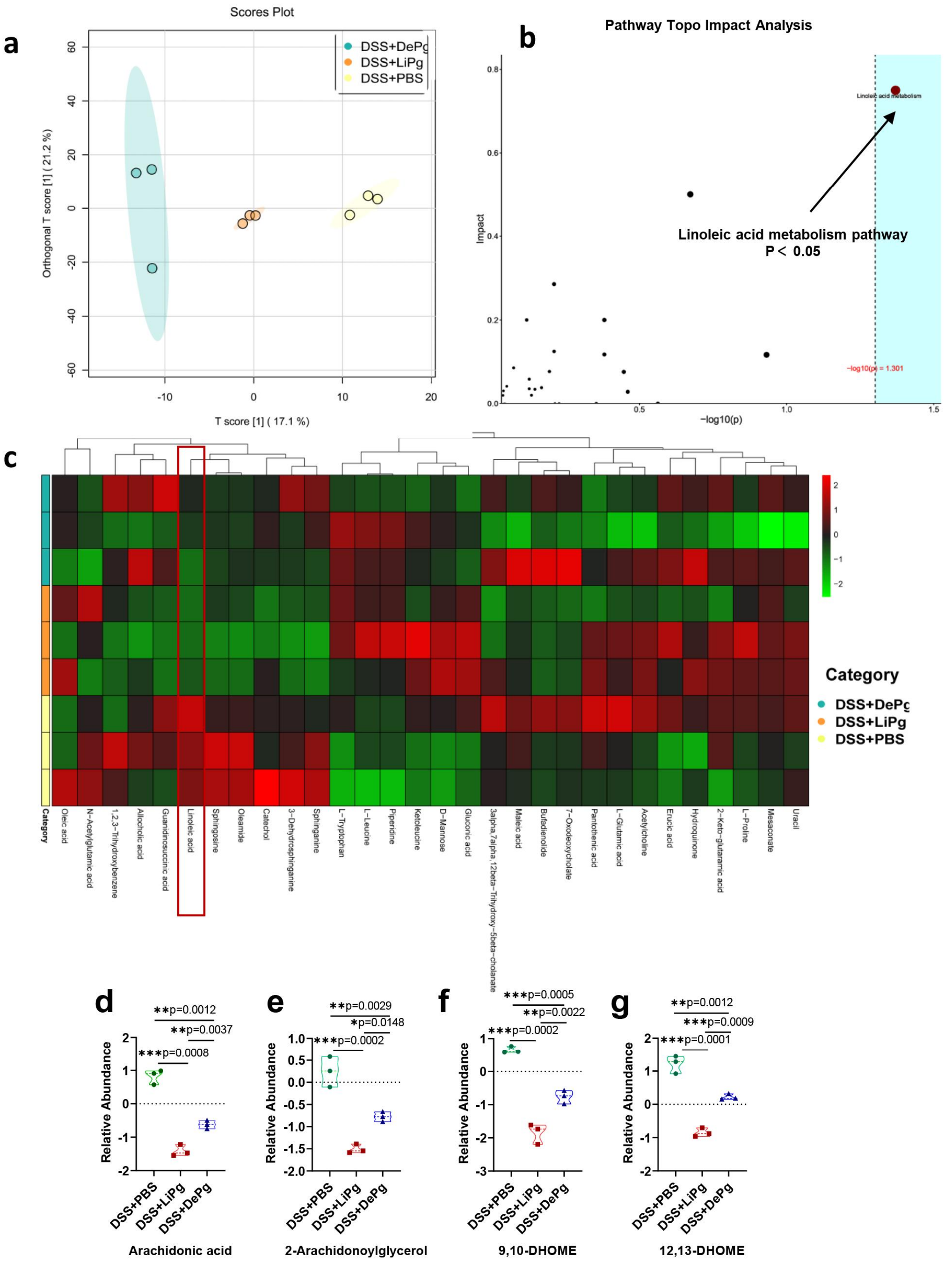

Supplementary Fig. 8. Oral administration of Pg led to a decrease in the levels of linoleic acid. (**a**) Supervised orthogonal partial least squares discriminant analysis (OPLS-DA) of DSS+PBS, DSS+LiPg, and DSS+DePg group metabolomes based on untargeted LC–MS/MS. n=3 independent samples. Different groups are marked with different colours, and the area marked by the ellipse is the 95% confidence area of the sample point. (**b**) Pathway Topo Impact Analysis was used to verify whether the linoleic acid pathway plays a key role in the biological process among DSS+PBS, DSS+LiPg, and DSS+DePg mice. The abscissa is the p value of the ORA analysis, and the blue area is significant ($p<0.05$). The ordinate is the topology analysis impact. (**c**) Top 30

thermograms of metabolite content. The vertical axis is sample grouping information—the horizontal axis indicates metabolites. The upper cluster tree is the similarity clustering of the distribution of metabolites in each sample, the left cluster tree is the sample cluster tree, the middle heatmap is the metabolite content, and the colour *versus* metabolite content (Z score) scale is shown in the upper right. (**d-g**) Histogram of metabolite differences upstream and downstream of the linoleic acid signalling pathway. ORA: Overrepresentation analysis. Source data are provided as a Source Data file.

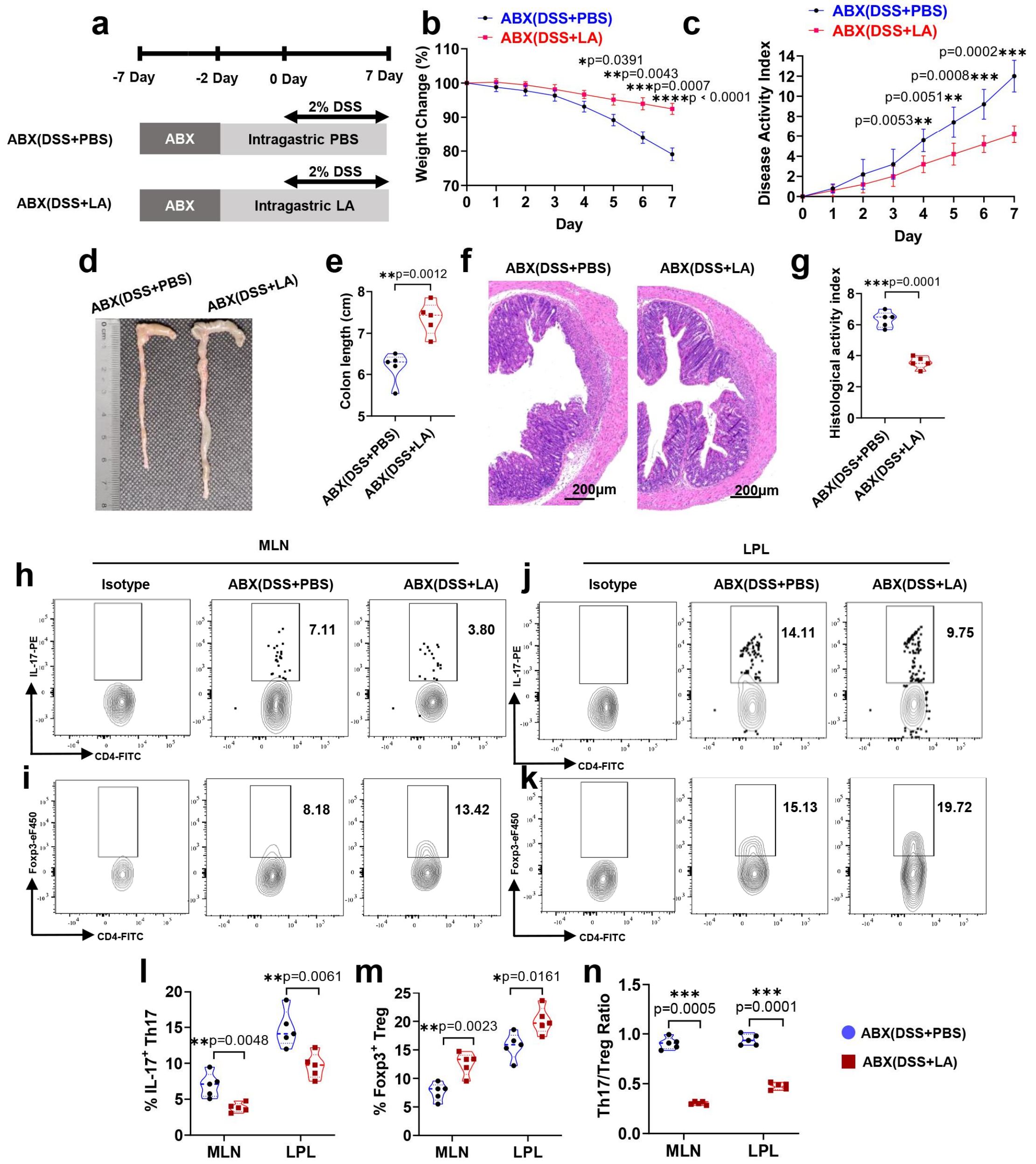

Supplementary Fig. 9. LA relieved colitis and decreased the Th17/Treg balance independent of the gut microbiota. (**a**) The experimental design. A DSS-induced colitis model was established. ABX: antibiotic cocktails intragastrically for five days for gut microbiota depletion. Receptor mice were gavaged with PBS (named ABX(DSS+PBS)) or linoleic acid (named ABX(DSS+LA)). (**b**) Body weight of ABX(DSS+PBS) and ABX(DSS+LA) mice during DSS-induced colitis, n=5 independent samples. (**c-g**) Disease activity index (DAI) (**c**), colon length (**d-e**), and histological score (**f-g**) of ABX(DSS+PBS) and ABX(DSS+LA) mice on day 8 after DSS induction, n=5 independent samples. Scale bar, 200 µm. (**h-k**) The proportions of IL-17^{+} Th17 cells and Foxp3^{+} Treg cells and their ratio among total CD4^{+} T cells within

the MLNs and LPLs were detected by flow cytometry and statistically analysed (**l-n**), n=5 independent samples. Data are presented as the mean ± SEM; *p<0.05; **p<0.01; ***p<0.001 by unpaired two-tailed t-test. MLN, mesenteric lymph node cells; LPL, colon lamina propria lymphocytes; DSS, dextran sodium sulfate. Source data are provided as a Source Data file.

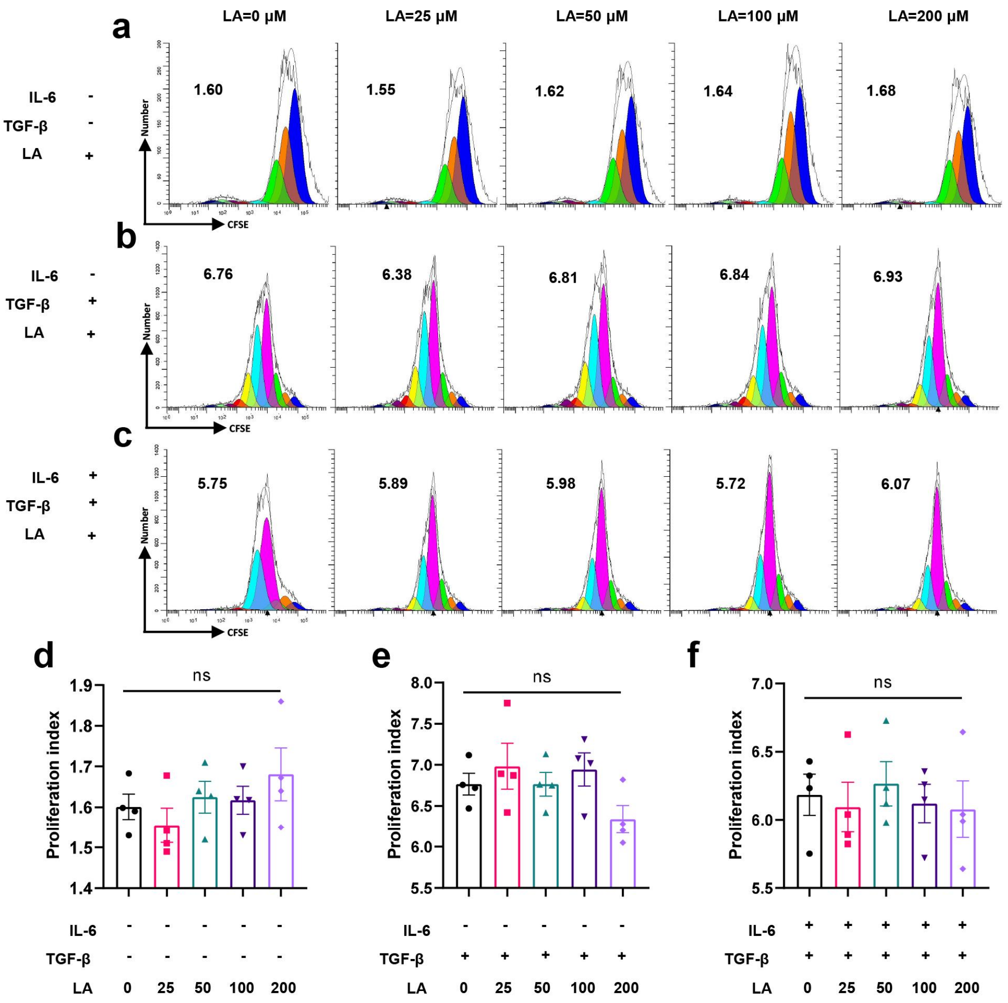

Supplementary Fig. 10. Effect of linoleic acid (LA) on the proliferation of CD4⁺ T cells into Th17 and Treg cells. (**a** and **d**) MACS-sorted naïve $CD4^+$ T cells were cultured with anti-CD3/CD28 beads for three days. The proliferation of $CD4^+$ T cells cocultured with different concentrations of LA was detected by flow cytometry and statistically analysed. (**b** and **e**) The proliferation of $CD4^+$ T cells induced with TGF-β under different concentrations of LA was detected by flow cytometry and statistically analysed. (**c** and **f**) The proliferation of $CD4^+$ T cells induced with TGF-β plus IL-6 under different concentrations of LA was detected by flow cytometry and statistically analysed. n=4 independent samples. Data are presented as the mean ± SEM; ns: no significant difference ($p > 0.05$) by two-tailed one-way ANOVA. Source data are provided as a Source Data file.

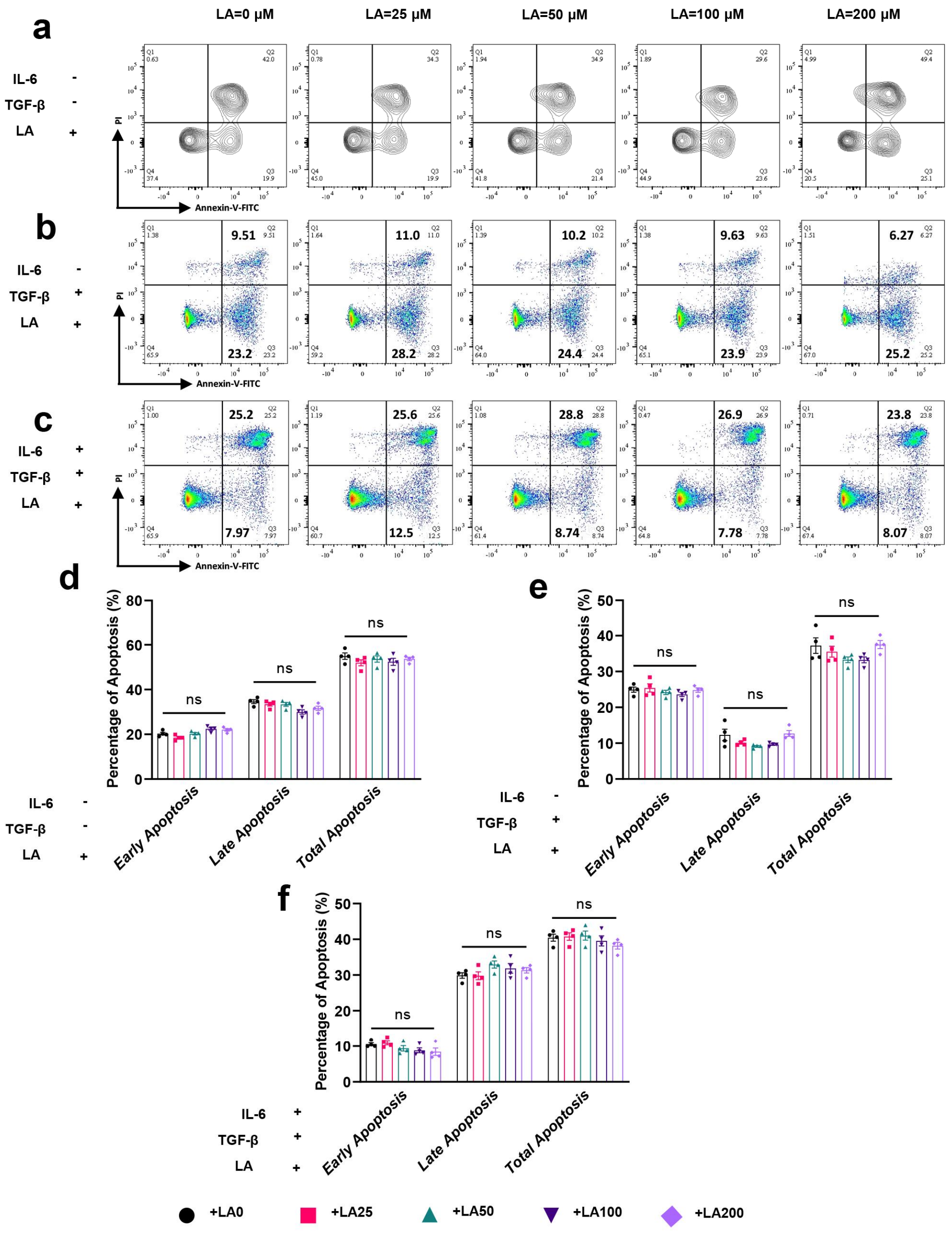

Supplementary Fig. 11. Effect of linoleic acid (LA) on the apoptosis of CD4^{+} T cells into Th17 and Treg cells. (**a** and **d**) MACS-sorted naïve CD4^{+} T cells were cultured with anti-CD3/CD28 beads for three days. The early, late, and total apoptosis rates of CD4^{+} T cells cocultured with different concentrations of LA were detected by flow cytometry and statistically analysed. (**b** and **e**) The early, late, and total apoptosis rates of CD4^{+} T cells induced with TGF-β under different concentrations of LA were detected by flow cytometry and statistically analysed. (**c** and **f**) The early, late, and total apoptosis rates of CD4^{+} T cells induced with TGF-β plus IL-6 under different concentrations of LA were detected by flow cytometry and statistically analysed. n=4 independent

samples. Data are presented as the mean ± SEM; ns: no significant difference ($p > 0.05$) by two-tailed one-way ANOVA. Source data are provided as a Source Data file.

a

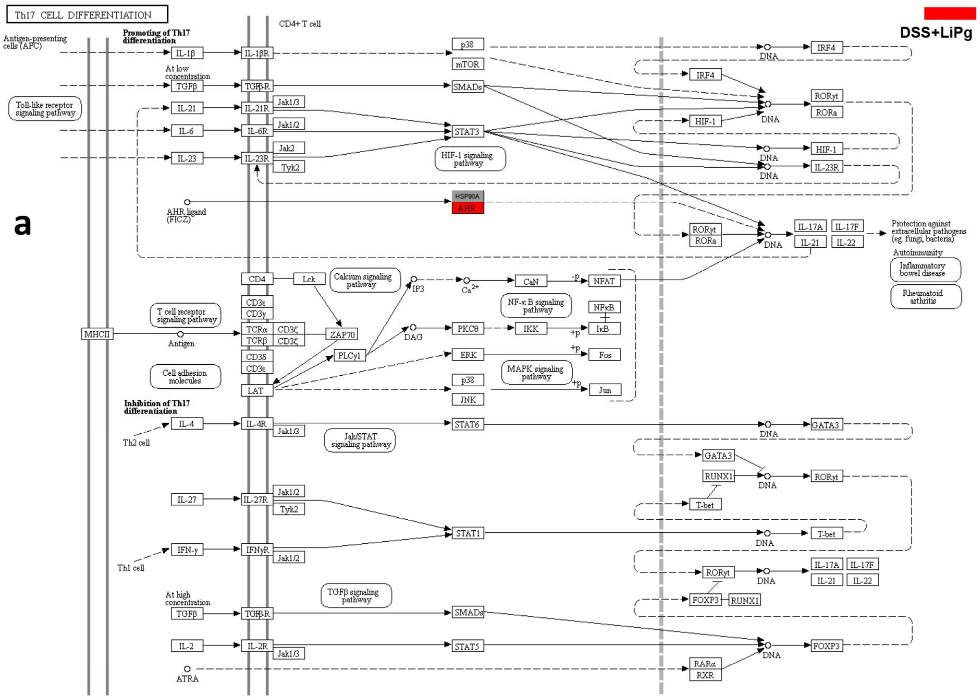

b

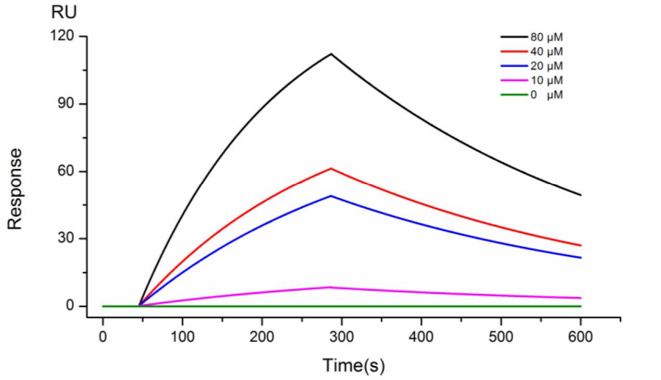

Concentration gradient binding curve of AHR and linoleic acid (LA)

Parameter	Paraphrase	Result
Ka($M^{-1}s^{-1}$)	association rate constant	3.94e1
Kd(s^{-1})	dissociation rate constant	2.62e-3
KD(M)	dissociation equilibrium constant (the affinity constant)	**6.64e-5**

Supplementary Fig. 12. The HSP90A and AHR complex was the characteristic gene biomarker of the DSS+LiPg group in the Th17 differentiation signaling pathway. (**a**) Based on the differential comparison between the KO (KEGG orthologous groups) LEfSe groups, the map pathway plots were coloured and the detected genes in the pathway maps and the gene biomarkers of each grouping were annotated. (**b**) LA was a ligand for AHR with a strong binding force. Concentration gradient binding curve of AHR and linoleic acid (LA) detected by localized surface plasmon resonance (LSPR). Source data are provided as a Source Data file.

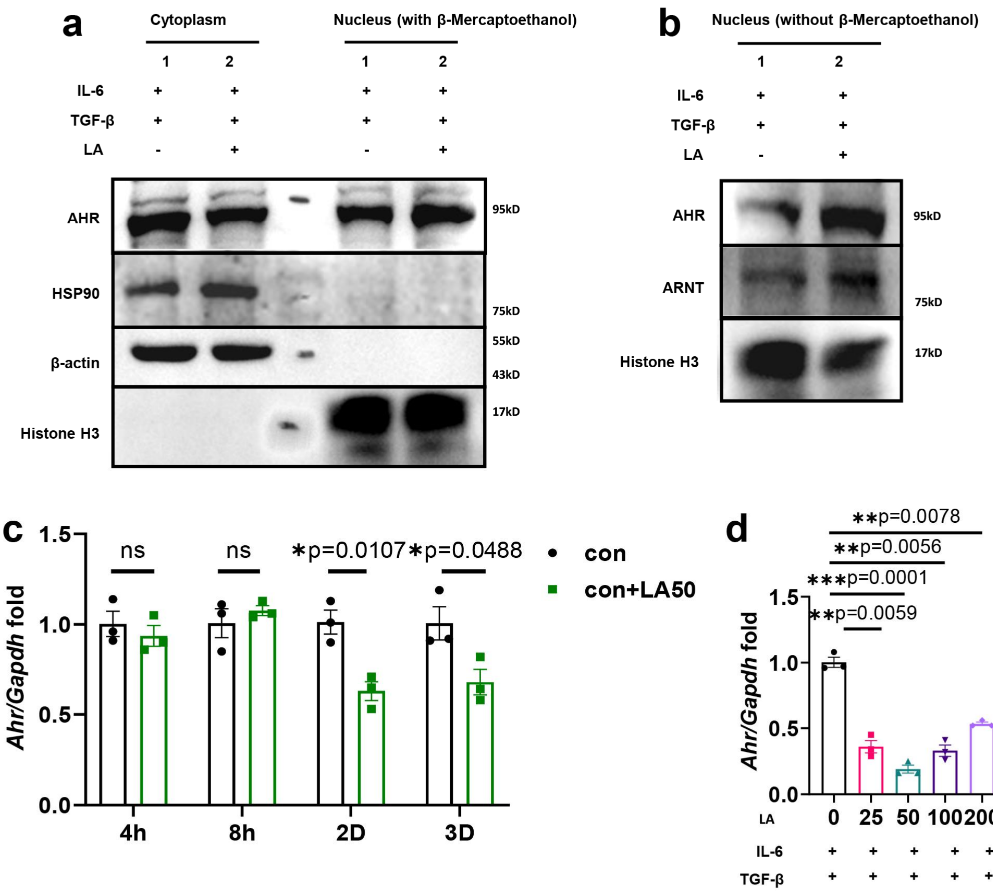

Supplementary Fig. 13. LA acts as an antagonist for AHR to repress the differentiation of Th17 cells. (**a**) WB detected changes in the subcellular translocation of AHR and HSP90 after isolating cytoplasmic and nucleoproteins. (**b**) The expression of AHR and AHR nuclear translocator (ARNT) in the nucleus was detected under non-reducing conditions after the extraction of nucleoproteins. Three times the experiment was repeated with similar results. (**c**) The expression of AHR after adding LA at different time points in Th17-inducing culture conditions. n=3 independent samples. *p<0.05; ns: no significant difference by two-tailed t-test. (**d**) The expression of AHR after adding different concentrations of LA on the 3rd day under Th17-polarizing conditions. n=3 independent samples. *p<0.05; **p<0.01; ***p<0.001 by two-tailed one-way ANOVA. Data are presented as the mean ± SEM. Source data are provided as a Source Data file.

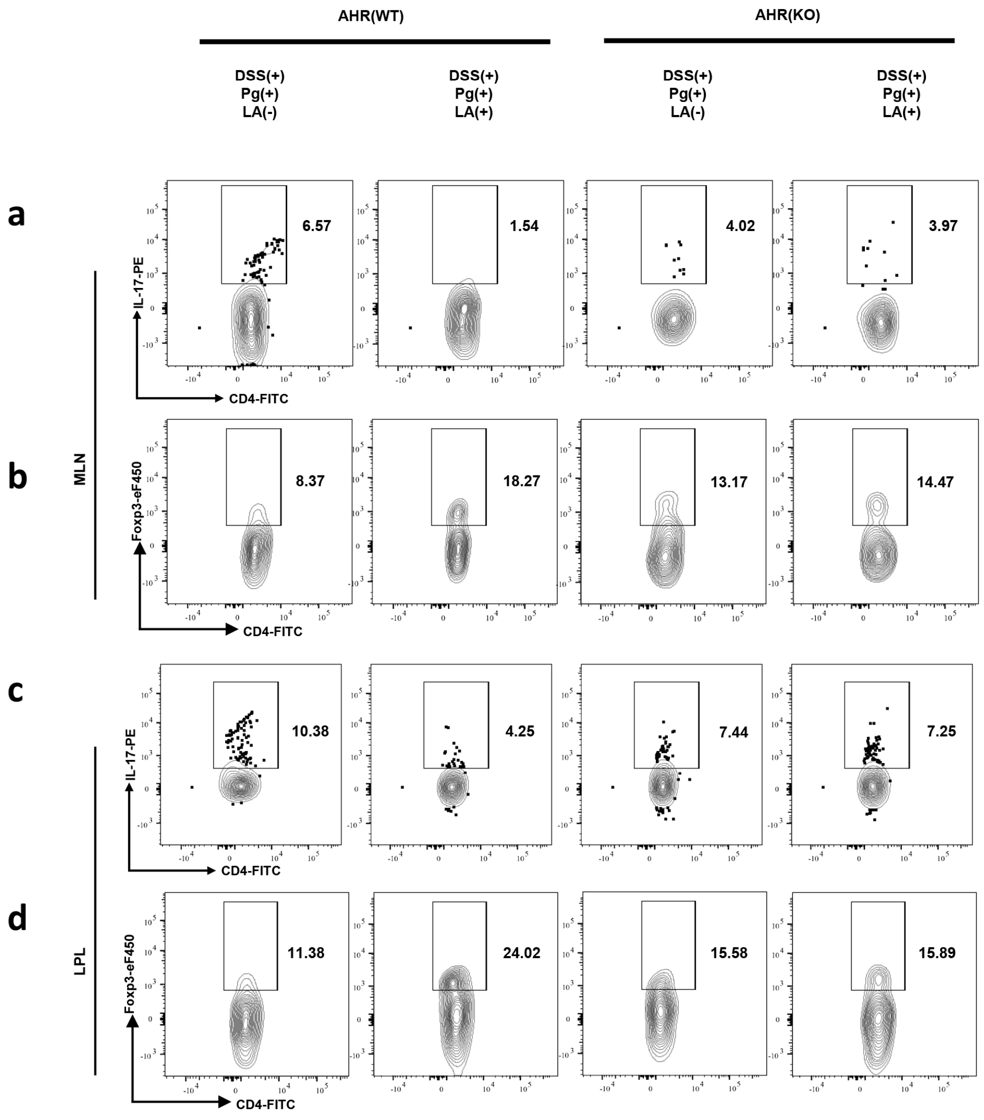

Supplementary Fig. 14. Oral administration of LA inhibited IL-17 and promoted Foxp3 in an AHR-dependent manner *in vivo*. **(a)** Representative flow cytometry of the the proportions of IL-17^{+} Th17 cells and **(b)** Foxp3^{+} Treg cells among total CD4^{+} T cells within the MLNs from the mice of the WT (DSS+Pg), WT (DSS+Pg+LA), KO (DSS+Pg), and KO (DSS+Pg+LA) groups. **(c)** Representative flow cytometry of the the proportions of IL-17^{+} Th17 cells and **(d)** Foxp3^{+} Treg cells among total CD4^{+} T cells within the LPLs from the mice of the WT (DSS+Pg), WT (DSS+Pg+LA), KO (DSS+Pg), and KO (DSS+Pg+LA) groups.Source data are provided as a Source Data file.

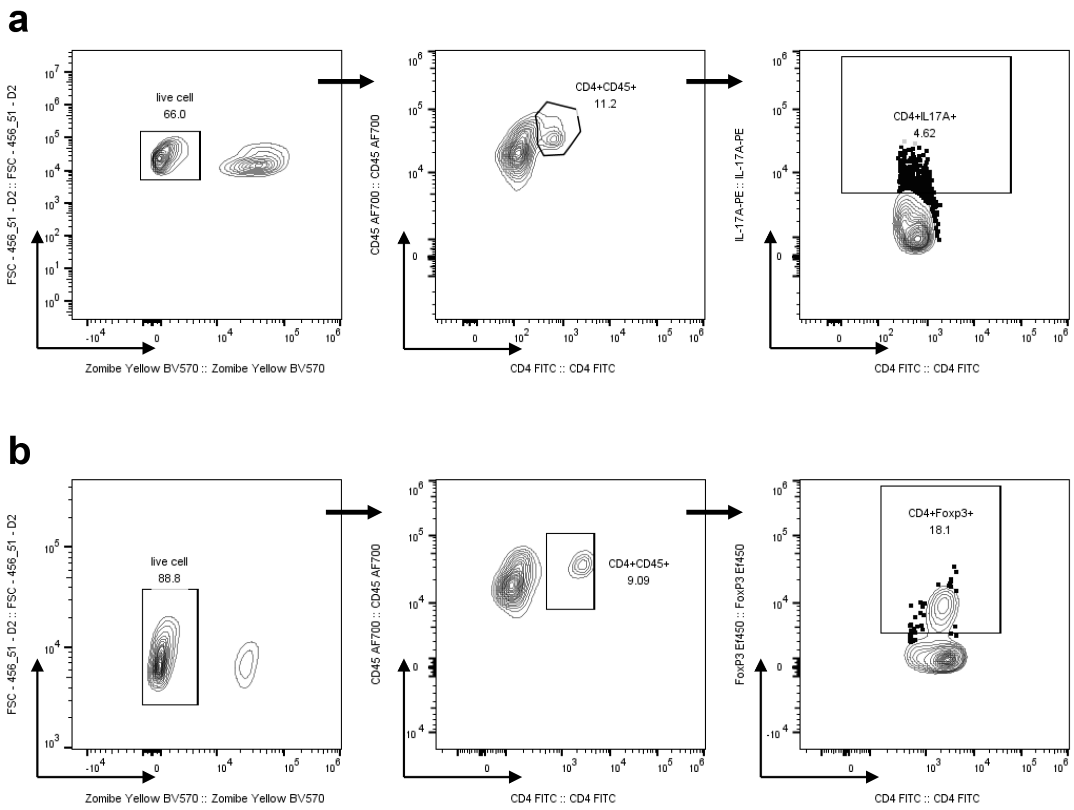

Supplementary Fig. 15. The gating strategies for the FACS analysis. (**a**) The gating strategies for the FACS analysis of the percentages of IL-17^{+}Th17 cells in MLNs and LPLs. (**b**) The gating strategies for the FACS analysis of the percentages of Foxp3^{+}Treg cells in MLNs and LPLs.

SUPPLEMENTARY TABLES

Supplementary Table 1. Probes Used in FISH

Probe target	Fluorophore	Sequence (5′→3′)	Reference
Bacteroidetes	Cy5	TCACGCGGCGTTGCTC	This study
P. gingivalis	Cy3	CAATACTCGTATCGCCCGTTATTC	(*1*)

Supplementary Table 2. Primers for Mouse Cytokine Analysis

Gene	Primer Bank ID	Forward primer (5′→3′)	Reverse primer (3′→5′)
Il-6	16193	TCTATACCACTTCACAAGTCGGA	GAATTGCCATTGCACAACTCTTT
Il-17a	16171	GGCCCTCAGACTACCTCAAC	TCTCGACCCTGAAAGTGAAGG
Il-10	16153	CTATGCTGCCTGCTCTTACTG	AACCCAAGTAACCCTTAAAGTC
Tfgb	21803	AACTTCTGTCTGGGACCCTG	CCGGGTTGTGTTGGTTGTAG
Il-22	50929	GACAGGTTCCAGCCCTACAT	TCGCCTTGATCTCTCCACTC
Foxp3	20371	AGAGCCCTCACAACCAGCTA	CCAGATGTTGTGGGTGAGTG
Rorc	19885	TGCAAGACTCATCGACAAGG	GGGGATTCAACATCAGTGCT
Stat1	20846	ACTGCACCCAAACCGAAGTC	TGGGGACACCTTTTAGCATCTT
Stat3	20848	CCCGTACCTGAAGACCAAGT	ACATCGGCAGGTCAATGGTA
Stat5	20850	CCAGCACGTTCATCATCGAG	TGTGCACATTCAGCTTTCCC
Ahr	11622	GCAGCTGTGTCAGATGGTGT	CTGAGCAGTCCCCTGTAAGC
Gapdh	14433	AGGTCGGTGTGAACGGATTTG	TGTAGACCATGTAGTTGAGGTCA

Supplementary Table 3. Antibodies used for WB

Antibodies	Source	Identifier	Dilution
Anti-Stat1 (phospho S727) antibody (EPR3146)	Abcam	Cat# ab109461	1:1000
Anti-Stat1 antibody (EPR23049-111)	Abcam	Cat# ab239360	1:1000
Anti-Stat5 (phospho S726) antibody (EPR1914(2))	Abcam	Cat# ab128896	1:1000
Anti-Stat1 (phospho Tyr701) antibody (D4A7)	CST	Cat# 7649T	1:1000
Anti-Stat5 antibody (D2O6Y)	CST	Cat# 94205T	1:1000
Anti-Stat5 (phospho Tyr694) antibody (D47E7)	CST	Cat# 4322T	1:1000
Anti-Stat3 (phospho Ser727) antibody (79D7)	CST	Cat# 9134T	1:1000
Anti-Stat3 (phospho Tyr705) antibody (D3A7)	CST	Cat# 9145T	1:1000
Anti-Stat3 antibody (124H6)	CST	Cat# 9139T	1:1000
Anti-beta Actin antibody (mAbcam 8224)	Abcam	Cat# ab8224	1:2000

Supplementary Table 4. Antibodies used for FACS

Antibodies	Source	Identifier	Dilution
Anti-mouse CD4 FITC (RM4-5)	eBioscience	Cat# 11-0042-82	1:200
Anti-mouse FoxP3 eFluor 450 (FJK-16s)	eBioscience	Cat# 48-5773-82	1:100
Rat IgG2a kappa Isotype Control eFluor 450 (eBR2a)	eBioscience	Cat# 48-4321-82	1:100
Anti-mouse IL-17a PE (eBio17B7)	eBioscience	Cat# 12-7177-81	1:100
Rat IgG2a kappa Isotype Control PE (eBR2a)	eBioscience	Cat# 12-4321-80	1:100
Anti-mouse CD45 AF700 (30-F11)	BioLegend	Cat# 103127	1:100
Zombie Yellow	BioLegend	Cat# 423104	1:300
PerCP/Cyanine5.5 anti-STAT1 Phospho (Ser727) (A15158B)	BioLegend	Cat# 686416	1:100
PerCP/Cyanine5.5 Mouse IgG1, κ Isotype Ctrl (MOPC-21)	BioLegend	Cat# 400149	1:100

SUPPLEMENTARY REFERENCE

1. Y. S. Choi, Y. C. Kim, K. J. Baek, Y. Choi, In Situ Detection of Bacteria within Paraffin-embedded Tissues Using a Digoxin-labeled DNA Probe Targeting 16S rRNA. *J Vis Exp*, e52836 (2015).
